# PI3K Functions Downstream of Cdc42 to Drive Cancer phenotypes in a Melanoma Cell Line

**DOI:** 10.1080/21541248.2023.2202612

**Published:** 2023-04-28

**Authors:** Rosemary Poku, Felix Amissah, Jamie K. Alan

**Affiliations:** a College of Medicine, Central Michigan University, Mt. Pleasant, MI, USA; b Department of Pharmaceutical Science, Ferris State University, Big Rapids, MI, USA; c Department of Pharmacology and Toxicology, College of Human Medicine, Michigan State University, East Lansing, MI, USA

**Keywords:** Cdc42, PI3K, Rho GTPases, melanoma

## Abstract

Rho proteins are part of the Ras superfamily, which function to modulate cytoskeletal dynamics including cell adhesion and motility. Recently, an activating mutation in Cdc42, a Rho family GTPase, was found in a patient sample of melanoma. Previously, our work had shown the PI3K was important downstream of mutationally active Cdc42. Our present study sought to determine whether PI3K was a crucial downstream partner for Cdc42 in a melanoma cells line with a BRAF mutation, which is the most common mutation in cutaneous melanoma. In this work we were able to show that Cdc42 contributes to proliferation, anchorage-independent growth, cell motility and invasion. Treatment with a pan-PI3K inhibitor was able to effectively ameliorate all these cancer phenotypes. These data suggest that PI3K may be an important target downstream of Cdc42 in melanoma.

## Introduction

The Ras superfamily of small GTPases are proteins that function as molecular switches to transmit intracellular signals initiated from extracellular stimuli [1]. Under normal biological conditions, Ras small GTPases are involved in many divergent cellular functions including cytoskeletal reorganization, cell survival, cell proliferation, transformation, and vesicular trafficking [1]. The Rho (Ras homologous) family of GTPases are members of the Ras superfamily that are best known for their role in modulating cytoskeletal dynamics [2]. The major Rho family members include Rho, Rac, and Cdc42, and these proteins are best known for their ability to regulate the actin cytoskeleton. Cdc42 also has a role in controlling cell polarity [2,3].

Under normal circumstances, small GTPases are tightly regulated molecular switches. When they are bound to GTP, they are activated. GTP binding allows a conformational change to engage effectors to elicit downstream signalling [4]. When GDP-bound, these proteins cannot engage effectors, and downstream signalling is terminated. Small GTPases also have intrinsic GTPase activity, which hydrolyzes GTP to GDP [4]. Positive modulators of Rho GTPases include GTP/GDP guanine nucleotide exchange factors (GEFs) which cause activation by facilitating the exchange of GDP for GTP [4,5]. Conversely, negative modulators such as GTPase accelerating proteins (GAPs) stimulate the hydrolysis of GTP and guanine nucleotide dissociation inhibitors (GDIs). GDIs inhibit GDP nucleotide dissociation and sequester Rho GTPases in the cytosol. Both actions lead to the inactivation of Rho GTPases [4,5].

Rho GTPases are essential for normal development and physiological processes [2]. In addition, they are also involved in many types of pathological processes, including cancer cell migration, invasion, and metastasis. Rho proteins are overexpressed in many cancer types [6,7]. Additionally, aberrant Rho protein activation manifests in various cancer subtypes by either upregulation of positive regulators (GEFs) and/or downregulation of negative regulators (GAPs, GDIs) [8]. Up until this point (with the notable exception of RhoH) [9], the central dogma was that misregulation of these proteins occurred by either overexpression or changes in their positive or negative regulators [6]. Recently, mutations in Rho GTPases in multiple cancer types have been identified and characterized, most notably the Rac1(P29S) mutation, which has been shown to be a driver mutation in melanoma [10,11]. Rac1(P29S) is a fast-cycling mutant that spends more time in the active GTP-bound state [11]. Rho GTPase function also requires posttranslational modification of the C-terminal membrane-targeting domain. Such lipid modification typically involves addition of a C15 (farnesyl) or a C20 (geranylgeranyl) to the CAAX box at the C-terminal end of the protein [12]. Cdc42 has a geranylgeranyl group added to its CAAX box, which is required for proper localization and function. In addition, there is typically a ‘secondary signal’ with the C-terminal hypervariable membrane targeting region [13]. In the case of Cdc42, there is a polybasic region that is important for membrane targeting and function [14,15].

While it only accounts for about 1% of skin cancer, melanoma carries the worst prognosis and is the most fatal skin cancer [16]. Melanoma can occur in several places in the body including the skin (cutaneous), mucous membranes (mucosal), and the eye (ocular). Cutaneous melanoma represents the majority of the cases (about 90%) [17,18] There are several driver mutations in signalling proteins that have been identified in melanoma, and their frequency varies depending on type of melanoma studies [10,11,17].

Melanomas frequently have either mutations in N-Ras or its downstream effector BRAF [17,19]. N-Ras mutations occur in about 15–20% of all non-uveal melanomas [17,19]. Mutations in N-Ras in melanoma typically occur at the Q61, the G12, or the G13 amino acids [1]. All these mutations result in an increase in GTP binding. The Rac1(P29S) mutant was first identified in melanoma samples by two seminal studies [10,11]. One of these studies also identified the Cdc42(G12V) mutation in melanoma samples [10]. The G12V mutation occurs at a conserved site, and the mutation locks the GTPase into the GTP-bound and active form. While any amino acid at the 12 position is partially activating, a valine at the 12 position is considered a fully activation mutation [20]. In addition to the G12V mutation found in melanoma sample exome sequencing, other mutations at the 12 and 13 position have also been identified. Cdc42(G12V) has been implicated as a driver mutation in melanoma necessary for invasion of melanoma cells [21,22] Furthermore, increased Cdc42 expression has been associated with a poorer prognosis and its effectors are associated with increased resistance to BRAF inhibitors [23,24]. Given this data, Cdc42 activity, both in the wild-type and constitutively active form, may be important for melanoma cell growth, migration, and invasion.

In its active GTP-bound state, Cdc42 is able to engage its downstream effectors to elicit biologic and oncogenic effects [3]. Many of Cdc42‘s effectors including p21-activated kinase (PAK), myotonic dystrophy kinase-related Cdc42-binding kinases (MRCKα and MRCKβ), activated Cdc42-associated kinase (ACK), Wiskott Aldrich Syndrome scaffold proteins (WASP and N-WASP), and the aPKC – Par6–Par3 complex, have been well characterized [23,25]. In fact, some of these effectors are overexpressed or have driver mutations that have been implicated in various cancer subtypes [23]. For example, ACK is overexpressed in NSCLC, ovarian cancer, HNSC, and endometrial carcinoma [26,27]. Additionally, several kinase-activating mutations in ACK have been found in und in ovarian cancer [26,27]. While much less characterized, Cdc42 can also activate phosphoinositol 3-kinase (PI3K). Though PI3K has been well-studied, its role as a Cdc42 effector is not well known. A study in *Saccharomyces cerevisiae* showed that the p85 unit of PI3K can couple to the effector domain of Cdc42 [22].

In our previous work, we identified PI3K as a protein that worked either downstream or in parallel to activated Cdc42(G12V) in a whole organism, *C. elegans* [28]. When expressed in the PDE neurons, Cdc42(G12V) in *C. elegans* it interacts with the actin cytoskeleton to produce a lamellipodial protrusion. In another study, we found that multiple components of the PI3K (the p85 and the P115 subunits) were able to suppress the lamellipodia protrusions driven by Cdc42(G12V) *in vivo*. The interaction of Cdc42(G12V) and PI3K was not specific to the PDE neurons, as we also found that knocking out PI3K subunits suppressed the activated Cdc42(G12V) phenotype in the VD/DD motor neurons as well [28].

In this work, we show that Cdc42(G12V) drives cancer phenotypes in BRAF mutant A375 melanoma cells, including proliferation, anchorage-independent growth, migration, and invasion. Furthermore, the use of a PI3K inhibitor can attenuate these phenotypes. Our work is the first to show that Cdc42(G12V) enhances the carcinogenic potential of melanoma cells that harbour a BRAF mutation, the most common mutation seen in melanoma. Furthermore, the use of a PI3K inhibitor in cells overexpressing Cdc42(G12V) can decrease proliferation, anchorage-independent growth, migration, and invasion of A375 melanoma cells. Our work is the first to show that target targeting PI3K may be an attractive option to attenuate activated Cdc42(G12V) signalling.

## Materials and methods

### Molecular constructs and human melanoma cells

Mammalian expression constructs for empty vector (pBabeHAII), haemagglutinin (HA) epitope-tagged human Cdc42 (wild-type, WT) and human Cdc42(G12V) were generously provided by Dr. Adrienne Cox. The vector has been previously described [29]. In this study, we used the ubiquitously expressed isoform of Cdc42. This Cdc42 sequences (wild-type and G12V) were cloned into the pBabeHaII vector using BamHi and EcoRI restriction enzymes. The human melanoma A375 cell line (ATCC CRL-1619IG-2) was purchased from the ATCC (American Type Culture Collection, Manassas, Virginia).

### Cell culture and transfection

A375 melanoma cells were grown in Dulbecco’s modified Eagle medium (Thermofisher, Rockford, IL) supplemented with 10% foetal bovine serum (FBS; Sigma, St. Louis, Missouri) and 1% penicillin-streptomycin (P/S) (‘complete culture medium’) and maintained in 5% CO_2_ at 37°C. A375 cells were transfected with expression constructs encoding vector only (VO) Cdc42 [30] or Cdc42(G12V) using Lipofectamine 3000 (Thermofisher, Rockford, IL) according to the manufacturer’s instructions. For selection of stably expressing cell lines, cells were grown in complete medium supplemented with 0.5 ug/ml puromycin (Sigma, St. Louis, Missouri) for 5 to 7 days, after which>50 colonies were pooled for use.

### Antibodies and immunoblot analysis

Cells were lysed in RIPA lysis buffer containing 1× protease inhibitor cocktail (Thermofisher, Rockford, IL). Lysates were cleared by centrifugation, and protein concentrations were determined using the BCA Protein assay (Thermofisher, Rockford, IL). Protein for each sample (50 µg), prepared in 2× Laemmli sample buffer, was resolved by using 12% SDS-PAGE. Proteins were transferred to polyvinylidene difluoride membranes (PVDF; Millipore, Burlington, MA), blocked at room temperature in 5% non-fat dry milk for 1 hour, and then probed for either Cdc42 (Cytoskeleton Inc., Denver, CO), pAKT (Cell ignalingS, Danvers, MA or AKT (Cell Signalling, Danvers, MA) for 24 h. Anti-β-actin (Cell Signalling, Danvers, MA was used as a loading control. Membranes washed in TBS-T were incubated in IRDye 680 Goat anti-mouse or IRDye 800 Goat anti-rabbit antibodies (LI-COR Bioscience, Lincoln NE), and fluorescence was analysed using a LI-COR Odyssey Fc imager (LI-COR Bioscience, Lincoln NE).

### Anchorage-independent growth transformation assay

Single-cell suspensions of transfected and non-transfected A375 cells (1×10^4^ cells per 6-well plate) were suspended in 0.4% agar (BD Biosciences, San Jose, CA) in complete medium with either DMSO or LY294002 (25 µM, Sigma Aldrich, St. Louis, MO) and layered on top of 0.6% agar as described previously [31]. After 21 days, colonies were stained with 3-(4,5-dimethylthiazol-2-yl)-2,5-diphenyltetrazolium bromide (MTT; Sigma). Colonies were counted using Image J software (https://imagej.nih.gov/ij/), and the average number colonies on triplicate dishes calculated.

### Cell proliferation assay

Cell proliferation assays were performed using MTT (Promega, Madison, WI) according to the manufacturer’s instructions. Cells were seeded in 96-well plates at a density of 2 × 10^3^ cells per well. After incubating the transfected and non-transfected cells for 0, 3, 5, and 8 days, cell proliferation was determined using the MTT assay (Promega, Madison, WI). The absorbance at 570 nm was recorded using Cytation 5 Multi-Mode Reader (BioTek, Winooski, VT). In another experiment, the cells were treated daily with either the vehicle (DMSO) or a PI3K inhibitor LY294002 (0–10 µM) for 2, 4, and 6 days, after which cell proliferation was determined using the MTT assay. Data representing eight replicates each of three independent experiments were analysed using GraphPad Prism 5.0.

### Cell migration assay

Wound healing assays were used to evaluate the migration ability of A375 cells stably expressing vector (VO), wild type Cdc42, or Cdc42(G12V). The cells were seeded at a density of 3.5 × 10^5^ cells/mL into each side of the Ibidi-cell culture inserts (Ibidi USA Inc., Fitchburg, WI), inserted in a 12-well cell culture plate and incubated (37°C/5% CO_2_) overnight. The cells were allowed to form two adherent confluent monolayers on either side of the tissue culture insert. The cells were then serum-starved by replacing the complete media with base media (DMEM) that did not contain serum, overnight. The inserts were gently removed to generate a ‘wound’ between the two confluent layers of cells. The monolayers were washed once with experimental media and then incubated with fresh experimental media containing the vehicle (DMSO) or a PI3K inhibitor, LY294002 (25 µM). Bright-field images of the ‘wound’ were captured using a Cytation 1 Cell Imaging Multi-Mode Reader (40× magnification) at every six hours until the control wounds had closed. The number of cells that migrated into the ‘wounds’ were counted, and the mean distance of migration was estimated using image J (https://imagej.nih.gov/ij/) for the control and treated cells. Data were analysed using GraphPad Prism 5.0.

### Cell invasion assays

The *in vitro* invasive potential of A375 cells transfected with either or vector (VO), wild type Cdc42, or Cdc42(G12V) was assessed using Matrigel matrix-coated invasion culture inserts (Corning, Bedford, MA), according to manufacturer’s instructions. Cell suspensions at a final density of 2.0 × 10^5^ cells/mL in DMEM medium containing 0.1% FBS were pretreated with either the vehicle or LY294002 (25 µM). The cell suspensions were added into each upper chambers of 24-well plate Matrigel invasion inserts (Corning, Bedford, MA) previously rehydrated with DMEM medium for 1 h at 37°C/5% CO_2_. Invasion inserts containing cell suspensions in media with 0.1% FBS were then carefully transferred into the wells of the 24-well plates containing media with 10% FBS. The plates were incubated for 24 h at 37°C/5% CO_2_ to allow the cells to invade from the upper chamber through Matrigel to the lower chamber of inserts. The non-invading cells on the upper chambers of inserts were removed using cotton swabs. The invading cells on the lower chambers of inserts were fixed with 3.7% formaldehyde in PBS and then stained with 1% crystal violet. Invading cells were imaged using a Leica DM5000B fluorescent microscope (Leica, Buffalo Grove, IL) fitted with a digital camera (DFC 480, Leica) at 100× magnification. The images were quantified using image j (https://imagej.nih.gov/ij/) software and analysed using GraphPad Prism 5.0.

### Immunoblotting

Cultured cells were seeded at 2 × 10^5^ cells per well in 6-well culture plates overnight. The cells were treated with PI3K inhibitor LY294002 (0–25 µM) for 24 h. The cells were lysed in ice-cold RIPA buffer (10 mM Tris-HCl, 150 mM NaCl, 1% sodium deoxycholate, 1% Triton X-100, and 0.1% SDS) containing protease inhibitors, centrifuged at 10,000×g for 5 min. The supernatant was collected and the protein in the lysate was quantitated using BCA Protein Quantification Kit (Thermo Fisher Scientific, Waltham MA), according to the manufacturer’s protocol. Protein samples were run on precast 4 to 20% gradient Tris-HCl gels (Bio-Rad, Hercules, CA) and then transferred onto polyvinylidene difluoride (PVDF) membranes (Bio-Rad, Hercules, CA). The membrane was then blocked with 5% non-fat dried milk in Tris-buffered saline (TBS) and Tween 20 (50 mM Tris-HCl pH 7.5, 150 mM NaCl, and 0.1% (v/v) Tween 20) for 1 h at room temperature and incubated with the appropriate specific primary antibody overnight at 4°C with gentle shaking. After multiple TBST washes, membranes were incubated with corresponding fluorescent-conjugated secondary antibody for 1 h at room temperature. Blots were visualized by fluorescence using a LI-COR Odyssey Fc imager (LI-COR Bioscience, Lincoln, NE).

### Statistical analysis

All results were expressed as the means ± SEM. Statistical significance was determined by either One-way ANOVA with Dunnett’s post-hoc test or by Student’s t-test as indicated in figure legends. P-values of less than 0.05 were considered statistically significant.

## Results

### Wild-type Cdc42 and Cdc42(G12V) promote A375 melanoma cell proliferation

**Figure 1.**
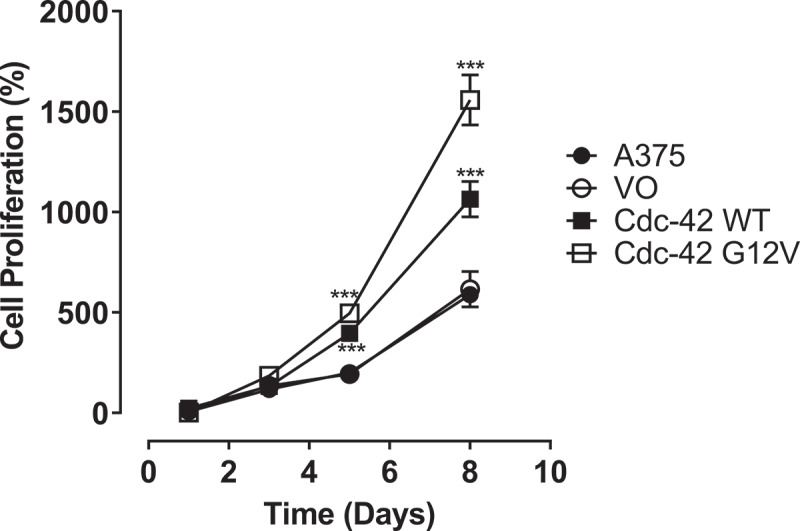
Wild-type Cdc42 and Cdc42(G12V) promote A375 melanoma cell proliferation in vitro. Cell proliferation assays were performed using untransfected A375 cells, and cell stably transfected with empty vector (VO), Cdc42 WT, or Cdc42(G12V). The percentage cell proliferation relative to the initial cells plated were quantified at 0 1,3,5, and 8 days using the MTT assay and analyzed using GraphPad prism. Significant differences compared with the parent A375 cells (***p<0.001) were determined by the Student’s t-test.

The Cdc42(G12V) mutation was identified in melanoma samples during whole exome sequencing of patient samples [10]. A mutation at position 12 alters the protein’s intrinsic GTPase ability, rendering the protein GTP-bound and active. Previous studies have found Cdc42 weakly transforming, and some researchers have observed that expression of Cdc42(G12V) is detrimental to cell growth [21,32]. However, with the discovery of the Cdc42(G12V) mutant in patient samples, and because Cdc42 expression results in a poorer prognosis, we hypothesized that Cdc42G12V may be important in melanoma cell growth, invasion and metastasis. We first sought to determine whether wild-type Cdc42 [30] and/or Cdc42(G12V) could increase proliferation in A375 melanoma cells. A375 cells contain the BRAF V600E mutation, which is the most frequently occurring mutation in human melanoma [32]. The data indicate a significant increase in the proliferation of the A375 cells stably expressing Cdc42 and Cdc42(G12V) compared to the other cell lines as shown in Figure 1. Although all the cell lines studied showed some increase in proliferation over the 8 day-period, the prominent increase in proliferation was observed in the cells expressing both wild-type Cdc42 Cdc42(G12V), which is consistent with the hypothesized characteristics of Cdc42 overexperssion and the G12V mutant phenotype.

### A PI3K inhibitor (LY294002) attenuates the levels of p-Akt in the A375 melanoma cell line, including cells expressing Cdc42(G12V)

**Figure 2.**
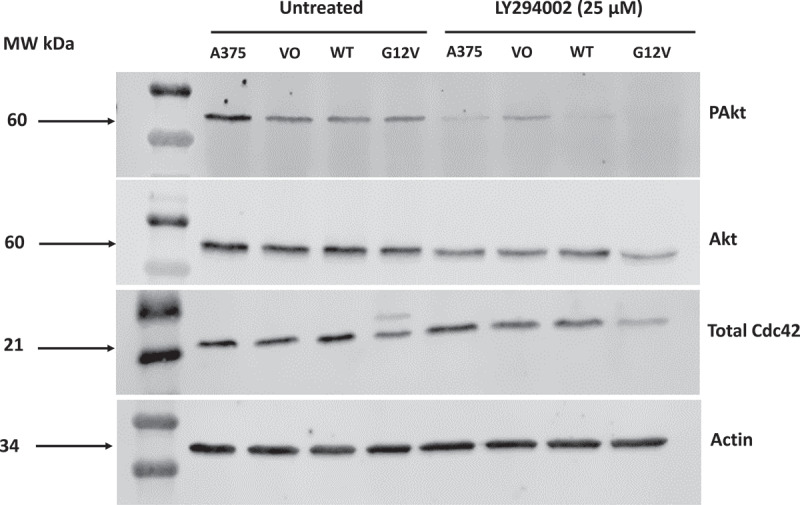
A PI3K inhibitor (LY294002) attenuates the levels of *p*-Akt in the A375 melanoma line, including cells expressing Cdc42(G12V). A375 cells or A375 cells stably expressing VO, Cdc42 WT or Cdc42(G12V) were treated with either DMSO (vehicle) or LY294002 (25 µM) for 24 h. Cells were analyzed by western blot for the differences in Akt, pAkt, and total Cdc42 expression in untreated and LY294002 treated cells. The blots show the levels of pAkt, Akt, and total Cdc42. Beta-actin was used as the loading control.

In our previous work in *C. elegans*, we sought to elucidate partners of activated Cdc42(G12V) in axon protrusion [28]. Similar to mammalian systems, in the *C. elegans* nervous system, Cdc42 activates the actin cytoskeleton [32]. In the dopaminergic PDE neurons expression of Cdc42(G12V) resulted in lamellipodia protrusions [32]. We were able to show that genetic knockout of PI3K and multiple components of the PI3K pathway were able to suppress the lamellipodia protrusions mediated by activated Cdc42 [32]. We reported that in *C. elegans*, PI3K works downstream or in parallel to Cdc42 to mediate the effects of activated Cdc42. In this work, we hypothesized that PI3K might be an important target downstream of activated Cdc42 in melanoma cells. In order to inhibit PI3K, we used the pan-PI3K inhibitor LY294002. As shown in Figure 2, at a concentration of 25 µM, we were able to observe significant inhibition of pAkt in all the cell lines tested (untransfected A375 cells, vector only, Cdc42(WT), and Cdc42(G12V)).

### Cdc42 and Cdc42(G12V) promote proliferation of A375 cells, which is attenuated by inhibition of PI3K

**Figure 3.**
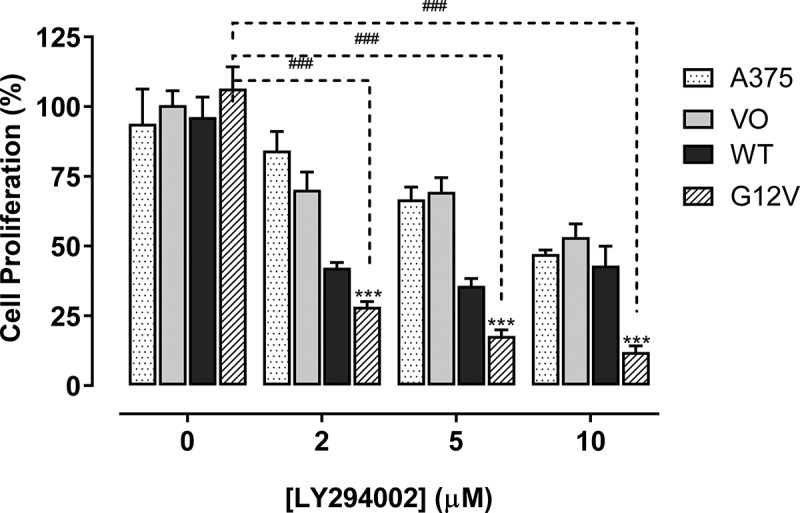
Cdc42(g12v) promotes the proliferation of A375 cells, which is attenuated with a PI3K inhibitor. Cell proliferation assays were performed using untransfected A375 cells and cells stably transfected with empty vector (VO), Cdc42 WT, or Cdc42(G12V). The cells were treated daily with either vehicle or a PI3K inhibitor LY294002 at concentrations of 0–10 µM. After 2 days, the percentage proliferation was analyzed using GraphPad prism. Significant differences compared with the parent A375 cells (^###^*p*<0.001) and compared with the different concentrations of LY294002 (***p<0.001) were determined by the Student’s t-test.

In our previous work, we found that PI3K was required downstream or in parallel of activated Cdc42(G12V) in *C. elegans* [28]. We hypothesized that inhibiting activated Cdc42 in melanoma cells by inhibiting PI3K could inhibit activated Cdc42-driven phenotypes such as proliferation, 3D cell growth, invasion, and migration. Because we found that both wild-type Cdc42 and Cdc4(G12V) greatly enhanced A375 melanoma cell proliferation (Figure 1), we wanted to see if we could decrease this growth after treatment with LY294002. As shown in Figure 3, we observed both concentration-dependent in the proliferation of all the cell lines used at 48 hours. The inhibitory effect was more significant in cells harbouring Cdc42(G12V) on day 2 (*p* < 0.001).

### Cdc42(g12v) enhances anchorage independent growth of A375 cells, and this is attenuated with inhibition of PI3K

**Figure 4.**
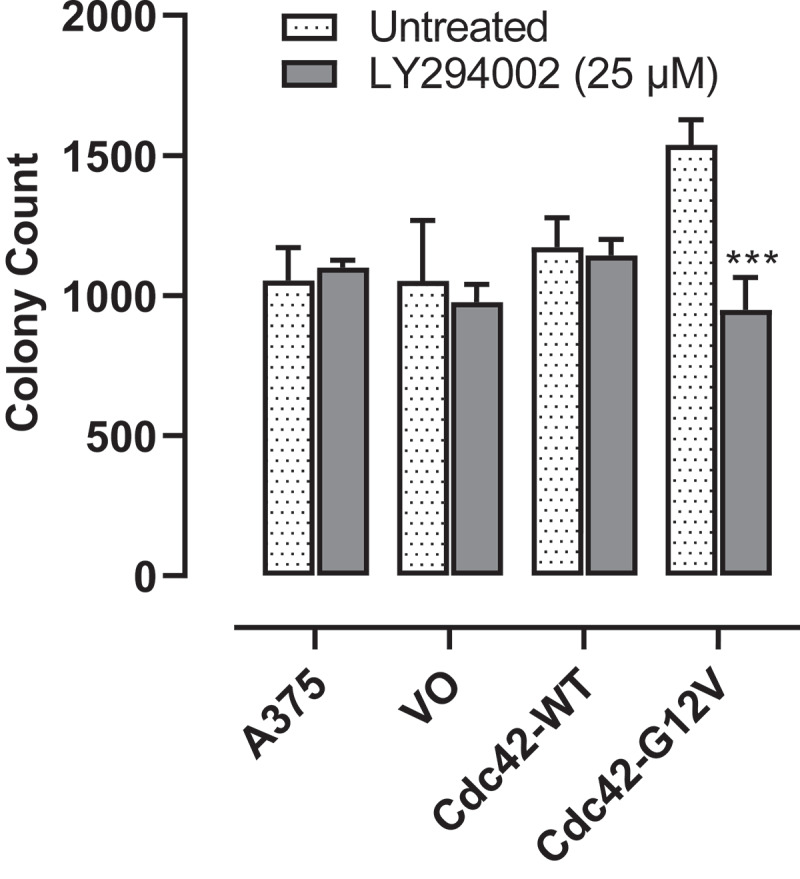
Cdc42 G12V enhances anchorage independent growth of A375 cells, and this growth is attenuated with inhibition of PI3K. A375 cells stably expressing VO, Cdc42 WT, and Cdc42(G12V) were plated in soft agar and treated weekly as described in the methods for 21 days. The number of colonies formed were counted using Image j, and the results are expressed as the means (± SEM, *n* = 4). Significance (***p< 0.001) was determined by the Student’s t-test.

While proliferation of cells is a hallmark of cancer, anchorage-independent growth, measured by a soft agar assay is also a measure of a cell’s tumorigenic potential. Therefore, we tested to see whether Cdc42 or activated Cdc42(G12V) was able to increase anchorage-independent growth in a BRAF mutant background. Indeed, Cdc42(G12V) greatly increased A375 anchorage-independent cell growth, however, wild-type Cdc42 expression did not result in a significant increase in the number of colonies. Next, we tested whether treatment with a PI3K inhibitor could attenuate the increase in anchorage-independent growth. As shown in Figure 4 treatment with LY294002 (25 µM) was able to significantly decrease the number of Cdc42(G12V) colonies (*p* < 0.001), suggesting that PI3K is important for activated Cdc42(G12V) anchorage-independent growth in BRAF-mutant melanoma.

### Cdc42(g12v) enhances migration of A375 cells, and this migration is attenuated by inhibition of PI3K

**Figure 5.**
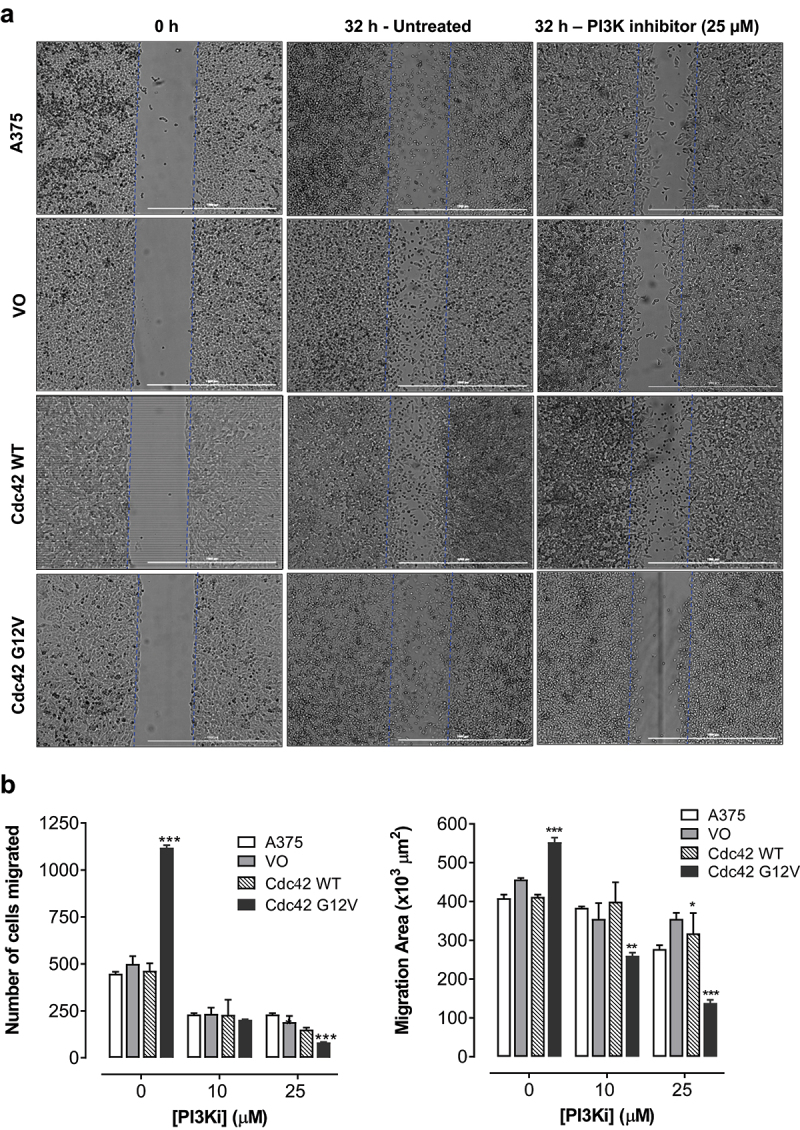
Cdc42 G12V promotes migration of A375 melanoma cells, and this migration is attenuated by inhibition of PI3K. a) Representative bright field images of confluent monolayers of A375, and A375 cells stably expressing VO, Cdc42 WT and Cdc42(G12V) treated with either vehicle control or PI3K inhibitor (LY294002, 25 µM) are shown. The blue lines indicate the demarcation of the original scratch wound. b) the distance of cell migration and the number of cells that migrated into the wound area are expressed as the mean (*n*=3) relative to the controls. The error bars represent the standard error of the mean. Significance (*p < 0.05, **p < 0.01, ***p < 0.001) was determined by the Student’s t-test.

Cell proliferation and anchorage-independent growth are more associated with primary features of a primary tumour. Because Cdc42 can induce changes in the actin cytoskeleton, we also wanted to look at other metastatic markers, such migration and invasion. These phenotypes are particularly important in melanoma because melanoma metastasis is associated with a dramatic drop in survival. First, we decided to look at migration making a ‘wound’ and then counting the number of cells that moved in to fill the space that was created. We found that activated Cdc42(G12V), but not wild-type Cdc42, significantly increased the number of cells that migrated into the wound area. Once we established that Cdc42(G12V) was able to increase the migration of melanoma cells, we wanted to investigate whether PI3K was required downstream of Cdc42(G12V) for this particular metastatic phenotype. Indeed, when we treated with a PI3K inhibitor, LY294002, the number of migrating cells were significantly reduced, suggesting that PI3K is important for this phenotype. Exposure to LY294002 (10 or 25 μM) inhibited both the area of cell migration and the number of migrated cells compared to the all the control untreated cell lines used in this study Figure 5a and b, respectively. The number of cells that migrated into the wounded area declined significantly after treatment with LY294002 (25 μM) in the cells overexpressing Cdc42(G12V) by 80.1 ± 2.8% compared to 55.8 ± 0.7% in the parent A375 cells, 50.6 ± 5.7% in the VO cells, and 54.7 ± 3.2% in Cdc42 [30] cells, respectively (Figure 5b). Also, the suppression of the area of migration was relatively more significant in the cells harbouring Cdc42(G12V) by 78.3 ± 1.6% compared to 25.8 ± 1.1% in the parent A375 cells, 17.6 ± 0.9% in the VO cells, and 8.7 ± 3.3% in Cdc42 [30] cells, respectively (Figure 5b).

### Cdc42(g12v) enhances invasiveness of A375 cells, and this invasion is attenuated with inhibition of PI3K

**Figure 6.**
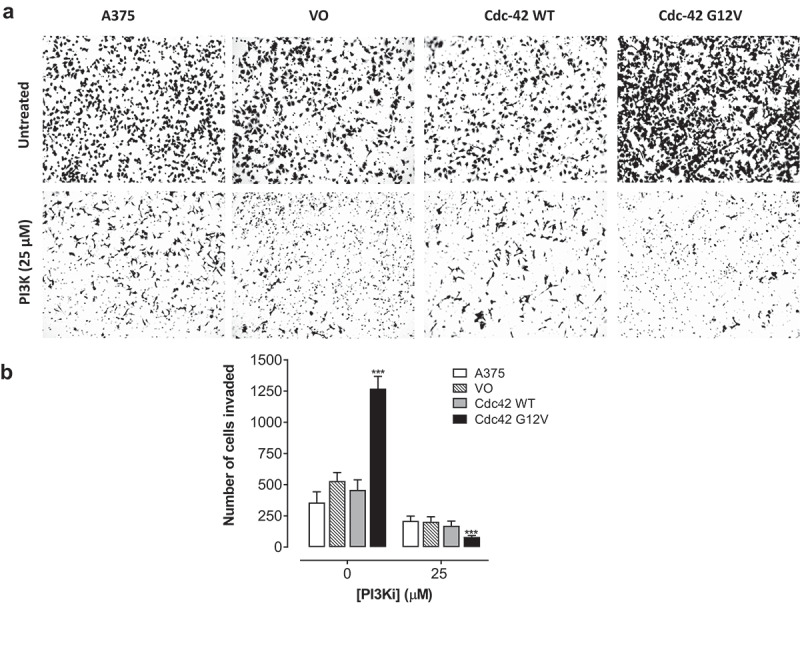
Cdc42(g12v) enhances the invasiveness of A375 cells, and this invasion is attenuated by inhibition of PI3K. A. A375 cells or A375 cells stably expressing VO, Cdc42 WT, or Cdc42(G12V) were treated with either DMSO (vehicle) or LY294002 (25 µM). a) Representative bright field images are shown. b) the number of invading cells were quantified using Image j. Values represent the mean ± SD of three independent experiments performed in triplicate. Significance (*p < 0.05; **p < 0.01; ***p < 0.001) compared with control group was determined by the Student’s t-test.

During metastasis, in addition to migration, cells invade through the extracellular matrix. To evaluate the invasive potential of Cdc42 and Cdc42(G12V) in BRAF mutant melanoma cells, we used a Matrigel invasion assay. The Matrigel recapitulates aspects of the extracellular matrix. When we quantified the number of cells that invaded through the Matrigel insert, we found that melanoma cells expressing Cdc42(G12V), but not Cdc42, migrated significantly more than the vector only control cells. When we treated the cells with LY294002, we found that inhibition of PI3K decreased the invasive properties of the Cdc42(G12V)-expressing cells (Figure 6). Exposure to LY294002 (25 μM) significantly inhibited invasion of the parent A375 cells by 35.8 ± 6.1%, the VO cells by 57.6 ± 3.9%, Cdc42 [30] cells by 58.7 ± 4.3%, and Cdc42(G12V) by 96.4 ± 1.2%, respectively, compared to their respective untreated control cells (Figure 6b). These data once again suggest that PI3K is necessary for the invasive potential for activated Cdc42(G12V) in BRAF mutant melanoma cells.

## Discussion

There have been many attempts to target Cdc42 and other small GTPases in human disease, particularly cancer. These strategies include targeting nucleotide binding, GEF interactions, effector binding, direct Cdc42 effectors, and Cdc42 membrane targeting [33]. Unfortunately, a significant number of these strategies have been ineffective. For example, it is difficult to create small molecules that can target and compete for nucleotide binding, because, unlike ATP, GTP binds with extremely high affinity [34]. Also, lack of specificity of GEF binding to the Rho GTPases makes it difficult to attain maximum efficacy without significant toxic effects [35]. To circumvent the myriad of challenges, we took a different approach. In a previous report, we found that multiple components of PI3K and the PI3K signalling cascade were required downstream of Cdc42(G12V) in multiple neuron types to induce characteristic phenotypes [28]. We concluded that PI3K is required downstream or in parallel to Cdc42 in *C. elegans* to induce an activated Cdc42 phenotype. The advantage of this type of approach is that it is done *in vivo*, and it considers genes that are both downstream and in parallel to the target of interest. We also found that other genes in the PI3K pathway were required to induce the activated Cdc42 phenotype, including AKT-1/2(Akt-1/2), RSKS-1(S6 kinase), and RICT-1(RICTOR). We hypothesized that PI3K might be an important target downstream of Cdc42(G12V) in melanoma. In addition to PI3K, we found other genes that suppressed activated Cdc42, including MIG-15(NCK-1 interacting kinase), ARX-4/7(Arp2/3 complex), WSP-1(WASP), WVE-1(WAVE), GEX-2/3(SCAR complex), UNC-34(ENAH), and TOCA-1(FNBP1). While beyond the scope of this work, these proteins may be useful and effective targets to test in melanoma cells in the future.

In this study, we present that Cdc42(G12V) overexpression in A375 melanoma cell lines, which have a background BRAF mutation, increased proliferation, anchorage independent cell growth, cell migration, and invasion, compared to the parent cell line Treatment of A375 cells expressing Cdc42(G12V) with PI3K inhibitor significantly inhibited proliferation, anchorage-independent cell growth, migration, and invasion in these cells compared to A375 parent cells, showing that PI3K is essential for Cdc42 activity in this context. The PI3K/AKT pathway is essential for cell proliferation, growth, survival, and migration [36]. This pathway is activated in human cancers and promotes abnormal cell growth and cancer cell transformation [37]. PI3K activates phosphatidyl inositol-4,5-bisphosphate (PIP2) through phosphorylation to phosphatidylinositol-3,4,4-trisphosphate (PIP3), which then phosphorylates Akt. Our findings are in agreement with our previous study in *C. elegans* which showed that PI3K is essential for activated Cdc42 activity [28]. Also in this study, a PI3K inhibitor also significantly decreased the expression of AKT and phosphorylated AKT in cells expressing Cdc42(G12V). This result suggests that the observed decrease in proliferation, invasion and migration seen with the treatment can be attributed to reduction in AKT activity.

Our study also sheds light on the interaction between Cdc42 and PI3K. Studies have shown PI3K as both an activator upstream of Cdc42 and an effector downstream of Cdc42, depending on context. For example, a study used the GTPyS-bound form of GST-Cdc42 in order to identify putative effectors of Cdc42 [38]. The authors found that an 85-kDa protein, the p85 subunit of PI3K, co-precipitated with GST-Cdc42. They also found that this interaction was detectable in multiple cell types, and a mutation in the effector domain (Cdc42Hs(T35A)) abolished this interaction. Another seminal study demonstrated that Cdc42 was able to bind directly to the p110 subunit of PI3K. The authors further showed that GTPγS-loaded Cdc42 strongly stimulated p110β kinase activity *in vitro*, and coexpression of constitutively Cd42 along with p110β/p85 in cells strongly increased p-Akt levels [39]. These studies suggest that PI3K is a direct downstream target of Cdc42. However, PI3K has also been identified as an upstream activator of Cdc42. In another study, the authors outlined a non-canonical signalling pathway where PI3K was responsible for activation of Cdc42 in neutrophils [40]. We found that inhibiting PI3K activity decreased Cdc42-driven proliferation, migration, and invasion. Because we were able to suppress even the constitutively activated Cdc42(G12V) protein when we inhibited PI3K, our study strongly suggests that PI3K is an effector of Cdc42 in this human melanoma cell line. However, the relationship between Cdc42 and PI3K is likely very complex, and the interactions may be to be cell-type specific. For example, a study showed a circular interaction between Cdc42 and PI3K. The authors identified a Cdc42 activation cycle that was regulated by PI3K downstream of the Fcγ Receptor (FcR) in macrophages. In this context, FcR activates Cdc42, which stimulates PI3K and actin polymerization. Then, a localized subsequent increase of 3′PIs inactivates Cdc42 to allow actin recycling, which is necessary for proper macrophage function [41].

In fact, the feedback loop described previously [41], may be the reason that activated Cdc42 (G12V) has been reported to be a putative tumour suppressor in the right circumstance [21]. Researchers have observed that Cdc42(G12V) overexpression can confer a growth disadvantage [21]. In fact, the transforming ability of Cdc42 may be dependent on its ability to cycle between its active GTP-bound and inactive GDP-bound states. An alternative hypothesis is that Cdc42 activity may fluctuate throughout the process of tumour initiation, development, maintenance and subsequent migration and metastasis. This idea is in line with our findings that wild-type increases cell proliferation and colony formation, but not anchorage-independent growth, migration, or invasion in this cell line. Interestingly, Cdc42 signalling has been implicated downstream of several tumour suppressors including PTEN and p53 [42–44]. Cdc42, in conjunction with Rho A, regulates PTEN localization and activity at the cell membrane, which contributes to the ability of PTEN to act as a tumour suppressor [44]. In this particular genetic background, we found that expression of Cdc42(G12V) enhanced cell proliferation, anchorage-independent growth, migration, and invasion. More work will be needed to characterize the role of Cdc42 activation in the context of other cell types. Additionally, with regards to the PI3K pathway, the role of circular signalling patterns that fluctuate in time and space may be important.

Taken together, the data from this study suggests that PI3K is an important effector protein downstream of activated Cdc42 in A375 human melanoma cell lines. Future studies will determine whether other identified effectors can be used as targets downstream of Cdc42 in melanoma cell lines. Additionally, our studies were done in a BRAF genetic background. We chose this genetic background, as this is the most common mutation in melanoma patients. Future studies will need to be done to determine whether Cdc42 and PI3K are important oncogenic targets in other melanoma genetic backgrounds. More work will be needed to characterize the role of Cdc42 activation in the context of other cell types. Additionally, with regards to the PI3K pathway, the role of circular signalling patterns that fluctuate in time and space may be important, and this concept should be further explored.

## References

[1] Wennerberg K, Rossman KL, Der CJ. The Ras superfamily at a glance. J Cell Sci. 2005;118(Pt 5):843–846. PubMed PMID: 15731001. DOI:10.1242/jcs.01660. 15731001

[2] Aspenstrom P, Fransson A, Saras J. Rho GTPases have diverse effects on the organization of the actin filament system. Biochem J. 2004;377(Pt 2):327–337. PubMed PMID: 14521508. DOI:10.1042/bj20031041. 14521508 PMC1223866

[3] Wennerberg K, Der CJ. Rho-family GTPases: it’s not only Rac and Rho (and I like it). J Cell Sci. 2004;117(Pt 8):1301–1312. PubMed PMID: 15020670. DOI:10.1242/jcs.01118. 15020670

[4] Geyer M, Wittinghofer A. Gefs, GAPs, GDIs and effectors: taking a closer (3D) look at the regulation of Ras-related GTP-binding proteins. Curr Opin Struct Biol. 1997;7(6):786–792. PubMed PMID: 9434896. DOI:10.1016/S0959-440X(97)80147-9. 9434896

[5] Olofsson B. Rho guanine dissociation inhibitors: pivotal molecules in cellular signalling. Cell Signal. 1999;11(8):545–554. PubMed PMID: 10433515. DOI:10.1016/S0898-6568(98)00063-1. 10433515

[6] Ridley AJ. Rho proteins and cancer. Breast cancer research and treatment. PubMed PMID: 14999150. 2004;84(1):13–19. DOI:10.1023/B:BREA.0000018423.47497.c6 14999150

[7] Alan JK, Lundquist EA. Mutationally activated Rho GTPases in cancer. Small GTPases. 2013;4(3): PubMed PMID: 24088985. DOI:10.4161/sgtp.26530 PMC3976972 24088985

[8] Ridley AJ, Hall A. The small GTP-binding protein rho regulates the assembly of focal adhesions and actin stress fibers in response to growth factors. Cell. 1992;70(3):389–399. PubMed PMID: 1643657. DOI:10.1016/0092-8674(92)90163-7. 1643657

[9] Preudhomme C, Roumier C, Hildebrand MP, et al. Nonrandom 4p13 rearrangements of the RhoH/TTF gene, encoding a GTP-binding protein, in non-Hodgkin’s lymphoma and multiple myeloma. Epub 2000/05/10PubMed PMID: 10803463 Oncogene. 2000;19(16):2023–2032. DOI:10.1038/sj.onc.1203521 10803463

[10] Hodis E, Watson IR, Kryukov GV, et al. A landscape of driver mutations in melanoma. PubMed PMID: 22817889 Cell. 2012;150(2):251–263. DOI:10.1016/j.cell.2012.06.024 22817889 PMC3600117

[11] Krauthammer M, Kong Y, Ha BH, et al. Exome sequencing identifies recurrent somatic RAC1 mutations in melanoma. Nature Genet. 2012;44(9):1006–1014. PubMed PMID: 22842228; PMCID: 3432702. DOI:10.1038/ng.2359. 22842228 PMC3432702

[12] Adamson P, Marshall CJ, Hall A, et al. Post-translational modifications of p21rho proteins. J Biol Chem. 1992;267(28):20033–20038. PubMed PMID: 1400319. DOI:10.1016/S0021-9258(19)88661-1. 1400319

[13] Michaelson D, Silletti J, Murphy G, et al. Differential localization of Rho GTPases in live cells: regulation by hypervariable regions and RhoGDI binding. J Cell Bio. 2001;152(1):111–126. PubMed PMID: 11149925. DOI:10.1083/jcb.152.1.111. 11149925 PMC2193662

[14] Adamson P, Paterson HF, Hall A. Intracellular localization of the P21rho proteins. J Cell Bio. 1992;119(3):617–627. PubMed PMID: 1383236. DOI:10.1083/jcb.119.3.617. 1383236 PMC2289677

[15] Shutes A, Berzat AC, Chenette EJ, et al. Biochemical analyses of the Wrch atypical Rho family GTPases. Methods Enzymol. 2006;406:11–26. PubMed PMID: 16472646. 16472646 10.1016/S0076-6879(06)06002-2

[16] Slominski A, Wortsman J, Carlson AJ, et al. Malignant melanoma. Epub 2001/09/26PubMed PMID: 11570904 Arch Pathol Lab Med. 2001;125(10):1295–1306. DOI:10.5858/2001-125-1295-MM 11570904

[17] Ch’ng S, Tan ST. Genetics, cellular biology and tumor microenvironment of melanoma. Epub 2009/03/11PubMed PMID: 19273108 Front Biosci(landmark Ed). 2009;14(3):918–928. DOI:10.2741/3286 19273108

[18] Teixido C, Castillo P, Martinez-Vila C, et al. Molecular markers and targets in melanoma. Cells. 2021;10(9):2320. Epub 2021/09/29PubMed PMID: 34571969; PMCID: PMC8469294. DOI:10.3390/cells10092320. 34571969 PMC8469294

[19] Ghosh P, Chin L. Genetics and genomics of melanoma. Epub 2010/02/04PubMed PMID: 20126509; PMCID: PMC2771951 Expert Rev Dermatol. 2009;4(2):131. DOI:10.1586/edm.09.2 20126509 PMC2771951

[20] Jaffe AB, Hall A. Rho GTPases: biochemistry and biology. Annu Rev Cell Dev Biol. 2005;21(1):247–269. PubMed PMID: 16212495. DOI:10.1146/annurev.cellbio.21.020604.150721. 16212495

[21] Stengel K, Zheng Y. Cdc42 in oncogenic transformation, invasion, and tumorigenesis. Epub 2011/04/26PubMed PMID: 21515363; PMCID: PMC3115433 Cell Signal. 2011;23(9):1415–1423. DOI:10.1016/j.cellsig.2011.04.001 21515363 PMC3115433

[22] Gadea G, Sanz-Moreno V, Self A, et al. DOCK10-mediated Cdc42 activation is necessary for amoeboid invasion of melanoma cells. Epub 2008/10/07PubMed PMID: 18835169 Curr Biol. 2008;18(19):1456–1465. DOI:10.1016/j.cub.2008.08.053 18835169

[23] Maldonado MDM, Dharmawardhane S. Targeting Rac and Cdc42 GTPases in Cancer. Epub 2018/06/03PubMed PMID: 29858187; PMCID: PMC6004249 Cancer Res. 2018;78(12):3101–3111. DOI:10.1158/0008-5472.CAN-18-0619 29858187 PMC6004249

[24] Svensmark JH, Brakebusch C. Rho GTPases in cancer: friend or foe? Oncogene. 2019;38(50):7447–7456. Epub 2019/08/21PubMed PMID: 31427738. DOI:10.1038/s41388-019-0963-7. 31427738

[25] Etienne-Manneville S, Hall A. Rho GTPases in cell biology. Nature. 2002;420(6916):629–635. PubMed PMID: 12478284. DOI:10.1038/nature01148. 12478284

[26] Mahajan K, Mahajan NP. ACK1/TNK2 tyrosine kinase: molecular signaling and evolving role in cancers. Oncogene. 2015;34(32):4162–4167. Epub 2014/10/28PubMed PMID: 25347744; PMCID: PMC4411206. DOI:10.1038/onc.2014.350. 25347744 PMC4411206

[27] Mahajan NP, Liu Y, Majumder S, et al. Activated Cdc42-associated kinase Ack1 promotes prostate cancer progression via androgen receptor tyrosine phosphorylation. Proc Natl Acad Sci USA. 2007;104(20):8438–8443. PubMed PMID: 17494760; PMCID: 1895968. DOI:10.1073/pnas.0700420104. 17494760 PMC1895968

[28] Alan JK, Struckhoff EC, Lundquist EA. Multiple cytoskeletal pathways and PI3K signaling mediate CDC-42-induced neuronal protrusion in C. elegans. Small GTPases. 2013;4(4):208–220. PubMed PMID: 24149939. DOI:10.4161/sgtp.26602. 24149939 PMC4011816

[29] Fiordalisi JJ, Johnson RL 2nd, Ulku AS, et al. Mammalian expression vectors for Ras family proteins: generation and use of expression constructs to analyze Ras family function. Methods Enzymol. 2001;332:3–36. Epub 2001/04/18PubMed PMID: 11305105. DOI:10.1016/s0076-6879(01)32189-4. 11305105

[30] Andoniou CE, Lill NL, Thien CB, et al. The Cbl proto-oncogene product negatively regulates the Src-family tyrosine kinase Fyn by enhancing its degradation. Mol Cell Biol. 2000;20(3):851–867. PubMed PMID: 10629042. DOI:10.1128/MCB.20.3.851-867.2000. 10629042 PMC85202

[31] Berzat AC, Buss JE, Chenette EJ, et al. Transforming activity of the Rho family GTPase, Wrch-1, a Wnt-regulated Cdc42 homolog, is dependent on a novel carboxyl-terminal palmitoylation motif. J Biol Chem. 2005;280(38):33055–33065. PubMed PMID: 16046391. DOI:10.1074/jbc.M507362200. 16046391

[32] Prefontaine GG, Walther R, Giffin W, et al. Selective binding of steroid hormone receptors to octamer transcription factors determines transcriptional synergism at the mouse mammary tumor virus promoter. J Biol Chem. 1999;274(38):26713–26719. PubMed PMID: 10480874. DOI:10.1074/jbc.274.38.26713. 10480874

[33] Tetley GJN, Murphy NP, Bonetto S, et al. The discovery and maturation of peptide biologics targeting the small G-protein Cdc42: a bioblockade for Ras-driven signaling. Epub 2020/01/22PubMed PMID: 31959628; PMCID: PMC7049977 J Biol Chem. 2020;295(9):2866–2884. DOI:10.1074/jbc.RA119.010077 31959628 PMC7049977

[34] John J, Sohmen R, Feuerstein J, et al. Kinetics of interaction of nucleotides with nucleotide-free H-ras p21. Biochemistry. 1990;29(25):6058–6065. Epub 1990/06/26. PubMed PMID: 2200519. DOI:10.1021/bi00477a025. 2200519

[35] Muller PM, Rademacher J, Bagshaw RD, et al. Systems analysis of RhoGEF and RhoGAP regulatory proteins reveals spatially organized RAC1 signalling from integrin adhesions. Epub 2020/03/24PubMed PMID: 32203420 Nat Cell Biol. 2020;22(4):498–511. DOI:10.1038/s41556-020-0488-x 32203420

[36] Xiao XH, Lv LC, Duan J, et al. Regulating Cdc42 and Its Signaling Pathways in Cancer: small Molecules and MicroRNA as new treatment candidates. Molecules. 2018;23(4):787. Epub 2018/03/30PubMed PMID: 29596304; PMCID: PMC6017947. DOI:10.3390/molecules23040787. 29596304 PMC6017947

[37] Rodgers SJ, Ferguson DT, Mitchell CA, et al. Regulation of PI3K effector signalling in cancer by the phosphoinositide phosphatases. Biosci Rep. 2017; 37(1):Epub 2017/01/14PubMed PMID: 28082369; PMCID: PMC5301276. DOI:10.1042/BSR20160432. PMC5301276 28082369

[38] Zheng Y, Bagrodia S, Cerione RA. Activation of phosphoinositide 3-kinase activity by Cdc42Hs binding to p85. J Biol Chem. 1994;269(29):18727–18730. Epub 1994/07/22. PubMed PMID: 8034624. DOI:10.1016/S0021-9258(17)32226-3. 8034624

[39] Fritsch R, de Krijger I, Fritsch K, et al. RAS and RHO families of GTPases directly regulate distinct phosphoinositide 3-kinase isoforms. Cell. 2013;153(5):1050–1063. PubMed PMID: 23706742. DOI:10.1016/j.cell.2013.04.031. 23706742 PMC3690480

[40] Chu JY, Dransfield I, Rossi AG, et al. Non-canonical PI3K-Cdc42-Pak-Mek-Erk Signaling promotes immune-complex-induced apoptosis in human neutrophils. Cell Rep. 2016;17(2):374–386. Epub 2016/10/06PubMed PMID: 27705787; PMCID: PMC5067281. DOI:10.1016/j.celrep.2016.09.006. 27705787 PMC5067281

[41] Beemiller P, Zhang Y, Mohan S, et al. A Cdc42 activation cycle coordinated by PI 3-kinase during Fc receptor-mediated phagocytosis. ?Mol Biol Cell. 2010;21(3):470–480. Epub 2009/12/04PubMed PMID: 19955216; PMCID: PMC2814791. DOI:10.1091/mbc.e08-05-0494. 19955216 PMC2814791

[42] Guo F, Zheng Y. Rho family GTPases cooperate with p53 deletion to promote primary mouse embryonic fibroblast cell invasion. Oncogene. 2004;23(33):5577–5585. Epub 2004/05/04PubMed PMID: 15122327. DOI:10.1038/sj.onc.1207752. 15122327

[43] Guo F, Zheng Y. Involvement of Rho family GTPases in p19Arf- and p53-mediated proliferation of primary mouse embryonic fibroblasts. Mol Cell Biol. 2004;24(3):1426–1438. Epub 2004/01/20PubMed PMID: 14729984; PMCID: PMC321455. DOI:10.1128/MCB.24.3.1426-1438.2004. 14729984 PMC321455

[44] Li Z, Dong X, Wang Z, et al. Regulation of PTEN by Rho small GTPases. Nat Cell Biol. 2005;7(4):399–404. Epub 2005/03/29PubMed PMID: 15793569. DOI:10.1038/ncb1236. 15793569

